# BRAF*^V600E^*-Informed TI-RADS Reclassification of Biopsy Eligibility in Thyroid Nodules: A Retrospective Oracle Decision–Simulation Study

**DOI:** 10.3390/diagnostics16193216

**Published:** 2026-10-04

**Authors:** Jiaojiao Ma, Xuehua Xi, Yang Li, Shanshan Wang, Ruyu Liu, Xuejiao Yu, Wanlin Huang, Mingyu Feng, Yong Cheng, Bo Zhang

**Affiliations:** 1 Department of Ultrasound, China-Japan Friendship Hospital, Beijing 100029, China; 2 National Center for Respiratory Medicine, State Key Laboratory of Respiratory Health and Multimorbidity, National Clinical Research Center for Respiratory Diseases, Institute of Respiratory Medicine, Chinese Academy of Medical Sciences, Department of Ultrasound, Center of Respiratory Medicine, China-Japan Friendship Hospital, Beijing 100029, China; 3 Department of Ultrasound, China-Japan Friendship Hospital, Chinese Academy of Medical Sciences & Peking Union Medical College, Beijing 100029, China; 4 Key Laboratory of Big Data-Based Precision Medicine, School of Engineering Medicine, Beihang University, Beijing 100191, China; 5 College of Information Science and Technology, Beijing University of Chemical Technology, Beijing 100029, China

**Keywords:** thyroid nodule, TI-RADS, BRAF*^V600E^*, fine-needle aspiration, machine learning

## Abstract

**Background/Objectives:** We evaluated a hypothetical oracle strategy that assumes BRAF*^V600E^* status is known before fine-needle aspiration (FNA), although testing usually requires FNA-derived material. **Methods:** This retrospective simulation included 4944 nodules from 4560 patients with linked ultrasound and molecular records following FNA evaluation. The cohort was selected for cytology and molecular testing and enriched for BRAF*^V600E^* positivity (42.4%) and TI-RADS 4C nodules (66.7%). The predefined rule combined BRAF*^V600E^*-positive upgrading with one-level downgrading of negative TI-RADS 4A/4B nodules. Outcomes were FNA-actionable classification changes and retention of originally FNA-actionable Bethesda V/VI nodules. Pre-FNA prediction was evaluated with patient-grouped cross-validation and training-fold calibration. **Results:** Under the hypothetical assumption of pre-FNA knowledge of BRAF*^V600E^* status, the oracle strategy reduced FNA-actionable classifications from 3454 to 2954 (net reduction 500; 14.5%) while retaining 1843/1920 Bethesda V/VI nodules (96.0%). In the selected surgical subgroup (365/4944), malignant-nodule retention was 189/196 (96.4%; 95% CI, 92.8–98.6%), with uncertainty extending below the exploratory 95% reference. Among 97 reclassified Bethesda III/IV nodules, only 2 had surgical pathology, and 1 was malignant. Patient-grouped random forest AUC was 0.693 (95% CI, 0.678–0.707). After calibration, a training-selected high-sensitivity threshold reduced classifications by 10.6%, with Bethesda V/VI retention of 95.6% (95% CI, 94.7–96.5%). **Conclusions:** This selected-cohort oracle simulation suggests a theoretical reduction in FNA-actionable classifications. Prospective evaluation in an unselected pre-biopsy population is required before clinical use.

## 1. Introduction

Thyroid nodules are increasingly detected with the widespread use of high-resolution ultrasound. Although improved imaging facilitates earlier diagnosis of clinically relevant thyroid cancers, it also increases the detection of small or biologically indolent lesions. Contemporary thyroid nodule management therefore aims to identify clinically meaningful malignancy while avoiding unnecessary invasive procedures [1].

Fine-needle aspiration (FNA) remains central to the diagnostic evaluation of thyroid nodules. However, broad application of ultrasound-based biopsy thresholds may increase the number of procedures performed for nodules that do not show high-risk cytology or that yield indeterminate results requiring repeat biopsy, molecular testing, or diagnostic surgery. Reducing FNA recommendations with limited diagnostic yield while maintaining detection of Bethesda V/VI cytology is therefore an important clinical objective [2,3,4,5,6,7,8].

TI-RADS provides a standardized ultrasound-based framework for malignancy risk stratification and FNA decision-making. By design, TI-RADS is based primarily on morphologic ultrasound features and nodule size, and it does not directly incorporate molecular tumor biology. As a result, some intermediate-risk nodules or nodules close to size-based biopsy thresholds may be categorized as FNA-actionable despite not yielding Bethesda V/VI cytology [2,3,4,5,6,7].

BRAF*^V600E^* is a common driver alteration in papillary thyroid carcinoma and reflects activation of the MAPK pathway. Molecular information from BRAF*^V600E^* status may therefore provide information complementary to ultrasound morphology. Nevertheless, BRAF*^V600E^* positivity should not replace sonographic assessment, and BRAF*^V600E^* negativity should not be interpreted as evidence of benignity, particularly because non-BRAF*^V600E^*-driven malignancies and follicular-patterned tumors may occur. Moreover, because BRAF*^V600E^* testing is commonly performed on FNA-derived material, its role in reducing initial FNA recommendations requires cautious evaluation [9,10,11,12].

Because BRAF*^V600E^* status is not normally available before the initial biopsy decision, we treated known BRAF*^V600E^* status as an oracle input: information unavailable at the intended decision point that provides an optimistic benchmark for this specified reclassification rule. This retrospective decision–simulation study quantified the difference between guideline-based TI-RADS eligibility and Bethesda V/VI cytology yield, estimated the hypothetical effect of molecular reclassification, examined cytology-specific and surgical-pathology outcomes, and evaluated pre-FNA clinical and ultrasound predictors of BRAF*^V600E^*. The oracle analysis does not establish the effect or safety of an available pre-biopsy test.

## 2. Materials and Methods

### 2.1. Study Design and Cohort

This retrospective nodule-level decision–simulation study included thyroid nodules evaluated between March 2019 and December 2025. Eligible nodules had linked ultrasound, FNA cytology records, and BRAF*^V600E^* mutation status, with sufficient ultrasound information for TI-RADS classification and reliable nodule-level matching. Nodules with incomplete key ultrasound or molecular data, uncertain target matching, duplicate records, or nonthyroid lesions were excluded. A cytology record was available for the included nodules, but 24 records lacked a classifiable Bethesda category; these nodules were retained in the classification simulation and reported separately, without contributing to the Bethesda V/VI retention denominator. Eligibility required prior FNA evaluation and molecular testing. Accordingly, the cohort is selected and enriched for BRAF*^V600E^* positivity (42.4%) and TI-RADS 4C nodules (66.7%); it does not represent an unselected population presenting for an initial biopsy decision.The study was approved by the Institutional Review Board of China-Japan Friendship Hospital. The requirement for informed consent was waived by the Institutional Review Board because of the retrospective design and the use of de-identified clinical data.

This study simulated how FNA-actionable classifications would have changed if BRAF*^V600E^* status had already been known and incorporated into TI-RADS-based decision-making at the time of the initial biopsy decision. Because this assumption does not reflect the usual temporal order of testing, in which BRAF*^V600E^* is determined from FNA-derived material, the simulation should be interpreted as an oracle upper-bound estimate, not as an evaluation of an implementable pre-biopsy rule or an actual prospective reduction in performed FNA procedures.

### 2.2. TI-RADS Classification and Guideline FNA Strategy

TI-RADS categories were assigned according to the guideline counting method. Suspicious ultrasound features included solid composition, marked hypoechogenicity, microcalcifications, irregular margins, and vertical orientation. Comet-tail artifact, when present in the absence of microcalcifications, was treated as a benign feature and subtracted from the suspicious-feature score [3].

The guideline-based TI-RADS strategy served as the reference strategy. TI-RADS 2 and 3 nodules were not recommended for FNA. FNA was recommended for TI-RADS 4A nodules larger than 15 mm and for TI-RADS 4B, 4C, and 5 nodules larger than 10 mm. FNA could be considered at lower size thresholds for nodules adjacent to the thyroid capsule or trachea: larger than 10 mm for TI-RADS 4A and larger than 5 mm for TI-RADS 4B, 4C, and 5. Nodules for which FNA was either recommended or could be considered were classified as FNA-actionable.

### 2.3. Oracle BRAF*^V600E^*-Informed TI-RADS Strategy

The rule assumed BRAF*^V600E^* status was already known at the initial biopsy decision. BRAF*^V600E^*-positive nodules were upgraded by one management level along the ordered categories 3, 4A, 4B, 4C, and 5, capped at category 5. BRAF*^V600E^*-negative TI-RADS 4A nodules moved to category 3, and negative TI-RADS 4B nodules moved to 4A. TI-RADS 4C and 5 nodules were not downgraded. The same category-, size-, and location-based FNA thresholds were then reapplied. The fixed rule was evaluated retrospectively in this cohort; predefinition does not establish clinical validity.

BRAF*^V600E^* negativity was not interpreted as evidence of benignity. It was used only as an auxiliary molecular modifier in the hypothetical rule for intermediate-risk nodules. The main upgrade/downgrade rule was defined before inspection of outcomes in this cohort. Its category transitions and size thresholds were not fitted or optimized against the observed Bethesda or surgical pathology outcomes.

### 2.4. Outcomes

The primary outcome was the change in the number and proportion of FNA-actionable nodules after BRAF*^V600E^*-informed reclassification. Net FNA reduction was the baseline actionable count minus the new actionable count, expressed relative to the baseline count. Gross reclassification to non-FNA status and newly actionable classifications were also reported separately. The principal cytological retention outcome was the proportion of guideline-actionable Bethesda V/VI nodules that remained actionable. Bethesda V/VI denotes suspicious-for-malignancy or malignant cytology and is not equivalent to histologically confirmed malignancy or freedom from clinically important missed cancer.

Secondary outcomes included the Bethesda V/VI proportion among FNA-actionable nodules, the number of missed original-FNA Bethesda V/VI nodules, the number of newly FNA-actionable Bethesda V/VI nodules, and Bethesda category-specific reclassification patterns.

AS-compatible status was used as a descriptive low-risk context within the non-FNA-actionable pathway and was not defined as the opposite of FNA-actionable status. AS-compatible nodules were defined as nodules 10 mm or smaller, not adjacent to the thyroid capsule or trachea, and without capsular protrusion or invasion in the available ultrasound records [13,14,15,16].

### 2.5. Machine Learning Analysis

We evaluated 14 pre-FNA predictors: age, sex, maximum nodule diameter, echogenicity, composition, homogeneity, margin, echogenic foci, halo, posterior acoustic features, Doppler flow, orientation, capsular relationship, and tracheal relationship. Bethesda cytology, pathology, TI-RADS category/score, FNA labels, other molecular variables, and patient identifiers were excluded from model inputs. Identifiers were used only for patient grouping and one-to-one nodule linkage. Audited BRAF*^V600E^* target labels were complete (2095 positive; 2849 negative), without target imputation [17,18].

Numeric predictors were median-imputed and standardized; categorical predictors used harmonized source categories and an explicit missing category. Recorded absence of echogenic foci or Doppler flow was retained as an observed category. Missing values occurred in age, echogenic foci, Doppler flow, capsular relationship, and tracheal relationship for 1, 1, 4, 3, and 3 nodules, respectively; the other predictors were complete. One additional capsular record coded Other/unclear was retained as such. Tree models used ordinal encoding with unseen categories assigned −1; logistic regression used one-hot encoding with unseen categories ignored. All learned preprocessing was fitted within the relevant training fold, including inner folds. No feature selection or synthetic oversampling was used.

Five models used the same stratified five-fold patient-grouped cross-validation: random forest, extra trees, gradient boosting, histogram gradient boosting, and logistic regression. All nodules from a patient remained in one-fold, and zero patient overlap was verified between training and test sets. Each outer-test fold contained 988–989 nodules from 911–913 patients, including 419 BRAF*^V600E^*-positive nodules. Hyperparameters were fixed for this analysis without a grid search, random search, or tuning against outer-test outcomes. Random forest remained the reference decision–simulation model, without reselection based on the highest grouped AUC. No external validation cohort was available.

Random forest and extra trees each used 500 trees, square-root feature sampling, a minimum of 5 samples per leaf, and balanced class weights. Gradient boosting used 100 trees, learning rate 0.1, maximum depth 3, and a minimum of 5 samples per leaf. Histogram gradient boosting used 100 iterations, learning rate 0.1, a maximum of 31 leaves, L2 regularization 1.0, and no early stopping. Logistic regression used L2 regularization (C = 1.0), the lbfgs solver, and a maximum of 2000 iterations. Other estimator settings used scikit-learn 1.9.1 defaults.

Within each outer training set, four-fold patient-grouped cross-validation generated random forest predictions for logistic recalibration, fitting an unpenalized logistic regression to logit probabilities (clipped to 0.000001–0.999999; lbfgs, maximum 2000 iterations). These inner predictions alone determined calibration and two finite ROC thresholds: maximum Youden’s index, or maximum specificity subject to BRAF*^V600E^* sensitivity of at least 90%. Selected calibrated thresholds ranged from 0.4243 to 0.4734 for Youden and 0.2994 to 0.3042 for the high-sensitivity target. The calibrator and thresholds were applied to untouched outer-test predictions from the model refitted on the full outer training set; zero patient overlap was also verified in all inner splits. Predicted labels were substituted into the identical rule in Section 2.3. The 90% training target does not guarantee test sensitivity or Bethesda V/VI retention, and these fold-specific thresholds are not a universal cutoff. A matched nodule-level random forest analysis used identical predictors, preprocessing, hyperparameters, and seed [17,18,19,20,21].

### 2.6. Statistical Analysis

Continuous variables were summarized as medians with interquartile ranges, and categorical variables as counts and percentages. Patient-cluster bootstrap resampling (1000 resamples, sampling patients with replacement and retaining all their nodules) estimated confidence intervals for the main oracle strategy because 375 patients (8.2%) contributed multiple nodules. Paired FNA-actionable classifications were compared using McNemar’s test. Exact Clopper-Pearson 95% confidence intervals were used for the larger surgical-pathology subgroup proportions; the capsular-invasion and lymph-node-metastasis subgroups were reported only as raw fractions because their actionable denominators were 4 and 1. For machine learning, AUC, average precision, Brier score, and logistic calibration intercept and slope were calculated from outer out-of-fold predictions. Sensitivity and specificity in the algorithm comparison were calculated at a fixed probability threshold of 0.50; decision simulations used the inner-training-selected thresholds. Patient-cluster bootstrap resampling (2000 resamples) estimated random forest AUC and decision-outcome confidence intervals and the paired AUC difference between grouped and nodule-level validation. These intervals condition on the generated out-of-fold predictions and do not include variability from repeating the entire model-fitting procedure. The prevalence-only Brier comparator assigned every nodule the overall cohort prevalence. An exploratory 95% retention reference was used for context; it was not a validated safety or noninferiority margin. A separate oracle sensitivity analysis restricted the reference set to formally FNA-recommended nodules.

The original oracle analyses used Python 3.11.7 (Python Software Foundation, Wilmington, DE, USA) and R 4.3.2 (R Foundation for Statistical Computing, Vienna, Austria). The patient-grouped machine-learning analysis used Python 3.12.14 (Python Software Foundation, Wilmington, DE, USA) and scikit-learn 1.9.1 (scikit-learn developers), with random seed 20260914 and inner-split seeds 20260915–20260919. The results describe simulated classification changes, not prospectively avoided FNA procedures. GenAI tools, specifically GPT-5.5 by OpenAI (OpenAI, San Francisco, CA, USA), assisted with language editing and statistical and figure-generation code under author supervision, as disclosed in the Acknowledgments.

## 3. Results

### 3.1. Study Population and Baseline Characteristics

A total of 4944 thyroid nodules from 4560 patients were included. The median patient age was 41.0 years (IQR, 34.0–53.0 years), and 3865 nodules (78.2%) occurred in female patients. The median maximum nodule diameter was 9.0 mm (IQR, 6.0–14.0 mm). BRAF*^V600E^* mutation was detected in 2095 nodules (42.4%). TI-RADS categories were 3 in 56 nodules (1.1%), 4A in 402 (8.1%), 4B in 1180 (23.9%), 4C in 3300 (66.7%), and 5 in 6 (0.1%). Baseline characteristics stratified by BRAF*^V600E^* status are shown in Table 1.

### 3.2. Guideline TI-RADS FNA Recommendation Burden

**Figure 1:**
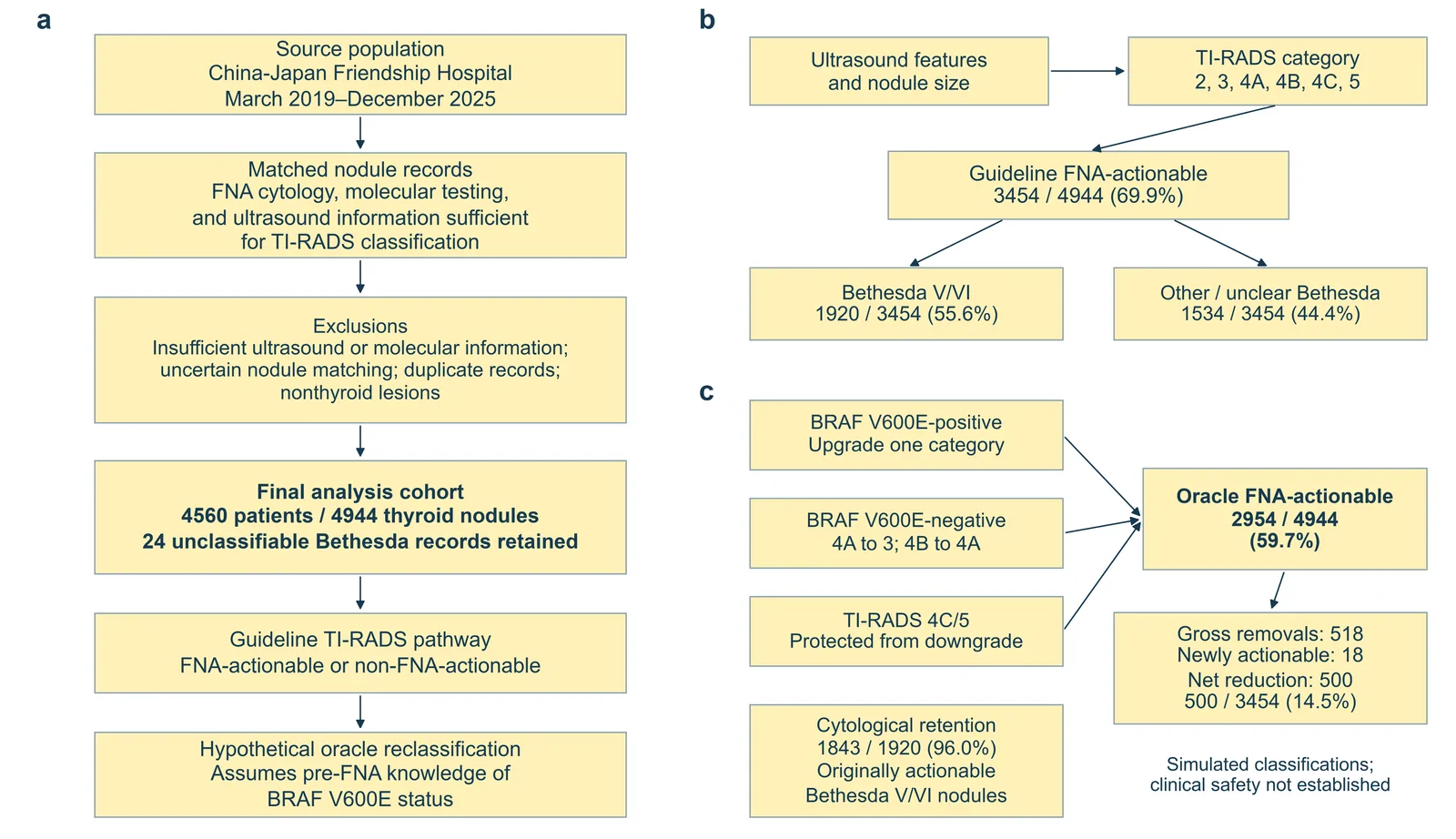
Study flow and hypothetical oracle pathway. (**a**) Cohort assembly; 24 records with unclassifiable Bethesda categories were retained. (**b**) Guideline FNA-actionable classifications and Bethesda V/VI composition. (**c**) BRAF*^V600E^*-positive upgrading, protection of TI-RADS 4C/5, and negative 4A/4B downgrading. Gross removals (518) minus newly actionable nodules (18) give a net reduction of 500/3454 (14.5%). Cytological retention does not establish clinical safety.

Under guideline-based TI-RADS, 3454 of 4944 nodules (69.9%) were classified as FNA-actionable, including 1813 nodules (36.7%) for which FNA was recommended and 1641 nodules (33.2%) for which FNA could be considered (Table 2; Figure 1). Among the 3454 FNA-actionable nodules, 1920 were Bethesda V/VI, corresponding to a Bethesda V/VI proportion of 55.6%. Conversely, 1534 FNA-actionable nodules (44.4%) did not yield Bethesda V/VI cytology, including 397 Bethesda III/IV nodules. These findings quantified the discordance between guideline-based FNA-actionable classification and high-risk cytology yield.

### 3.3. FNA Classification Reduction and Bethesda V/VI Retention

**Figure 2:**
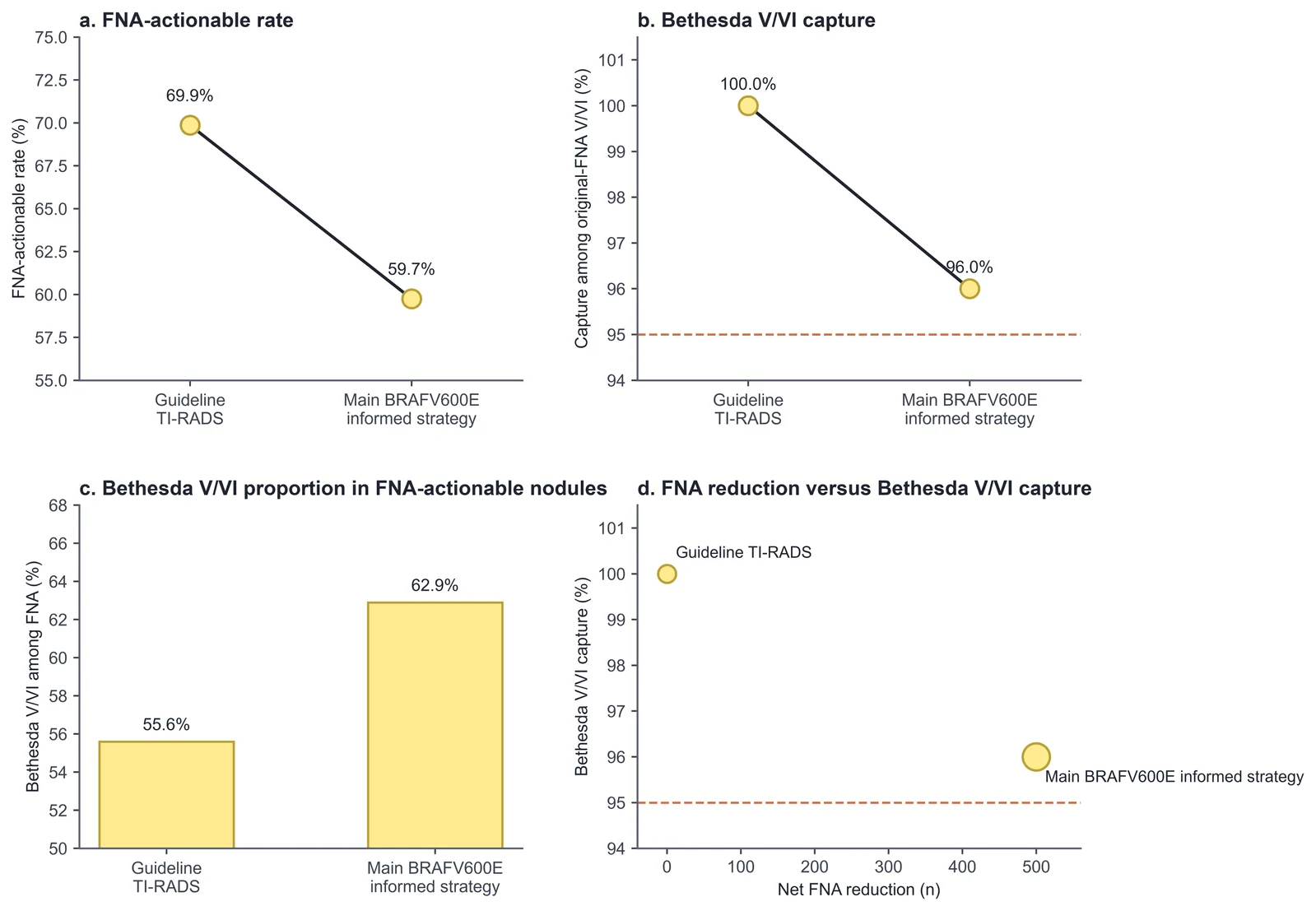
Simulated FNA classification changes and Bethesda V/VI retention. (**a**) FNA-actionable rate; (**b**) Bethesda V/VI retention among originally actionable nodules; (**c**) Bethesda V/VI proportion among actionable nodules; (**d**) the relationship between net FNA reduction and Bethesda V/VI retention. The dashed 95% line is an exploratory reference, not a validated safety or noninferiority margin. The oracle results assume pre-FNA knowledge of BRAF*^V600E^* status.

**Figure 3:**
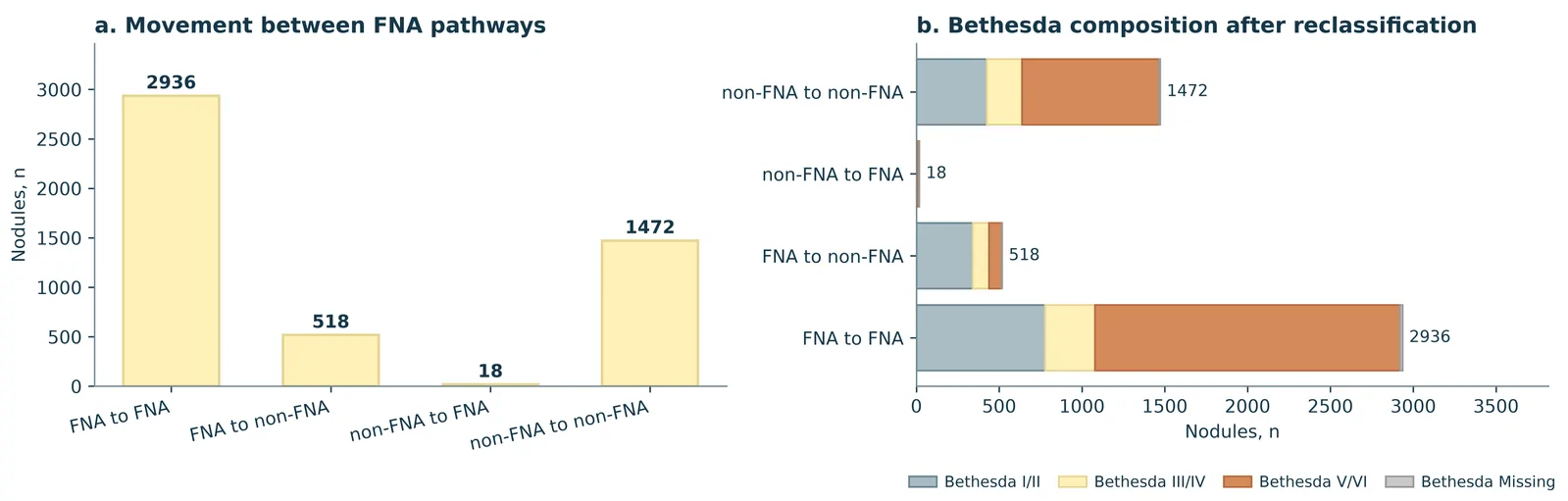
Reclassification flow after BRAF*^V600E^*-informed TI-RADS strategy. The figure shows movement between FNA-actionable and non-FNA-actionable pathways after BRAF*^V600E^*-informed reclassification, with Bethesda cytology composition displayed for retained, reclassified, and newly FNA-actionable nodules. (**a**) Movement of nodules between the FNA-actionable and non-FNA-actionable pathways after BRAF*^V600E^*-informed reclassification; (**b**) Bethesda cytology composition within each movement category shown in (**a**).

The main (oracle) BRAF*^V600E^*-informed strategy decreased FNA-actionable classifications from 3454 to 2954, corresponding to a net reduction of 500 classifications and a 14.5% relative reduction. In total, 518 originally FNA-actionable nodules were reclassified as non-FNA-actionable, whereas 18 nodules originally outside the FNA-actionable pathway became newly FNA-actionable (Table 2; Figure 2 and Figure 3). Among Bethesda V/VI nodules originally classified as FNA-actionable under guideline-based TI-RADS, 1843 of 1920 were retained after BRAF*^V600E^*-informed reclassification, yielding a Bethesda V/VI retention rate of 96.0%. The Bethesda V/VI proportion among FNA-actionable nodules increased from 55.6% to 62.9%. Using patient-cluster bootstrap resampling to account for the 375 patients (8.2%) who contributed more than one nodule, the absolute reduction in FNA-actionable classifications was 10.1 percentage points (95% CI, 9.2–11.0), the relative reduction was 14.5% (95% CI, 13.2–15.8%), and Bethesda V/VI retention was 96.0% (95% CI, 95.1–96.9%); the change in FNA-actionable status was significant (McNemar test, *p* < 0.001).

Seventy-seven original FNA-actionable Bethesda V/VI nodules were reclassified to the non-FNA-actionable pathway, whereas 14 Bethesda V/VI nodules originally outside the FNA-actionable pathway were newly classified as FNA-actionable. Overall, the number of FNA-actionable nodules decreased, while the proportion of Bethesda V/VI cytology among FNA-actionable nodules increased.

The 77 missed Bethesda V/VI nodules were concentrated in TI-RADS 4B (57/77) and 4A (20/77), were mostly 0.6–1.5 cm (61/77), and 70/77 (90.9%) were adjacent to the thyroid capsule or trachea. Most retained individually suspicious ultrasound features despite reclassification, including low echogenicity (68/77, 88.3%), solid composition (57/77, 74.0%), and irregular margin (51/77, 66.2%); these nodules were reclassified because of the restricted BRAF*^V600E^*-negative TI-RADS 4A/4B downgrade rule and size/location thresholds, not because they appeared sonographically reassuring.

### 3.4. Bethesda Category-Specific Reclassification

**Figure 4:**
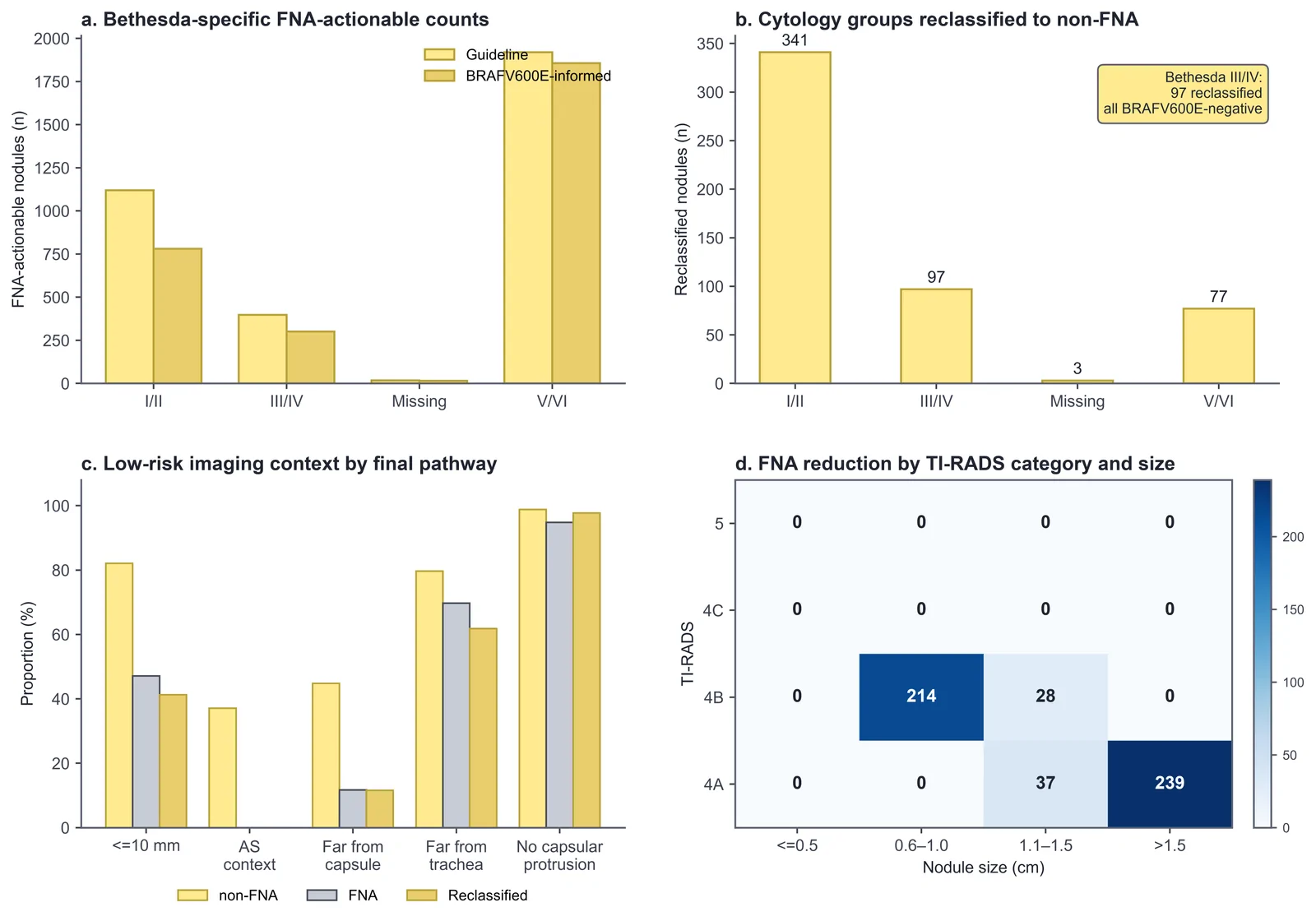
Bethesda-specific reclassification and AS-compatible context. The figure summarizes cytology groups contributing to FNA reduction and describes imaging features of nodules in the FNA-actionable and non-FNA-actionable pathways. AS-compatible status is a descriptive low-risk context, not the opposite of FNA. (**a**) Bethesda-specific FNA-actionable counts under guideline versus BRAF*^V600E^*-informed strategies; (**b**) cytology groups reclassified from FNA-actionable to non-FNA-actionable, by Bethesda category; (**c**) proportion of nodules with low-risk imaging features by final pathway (non-FNA, FNA, reclassified); (**d**) nodules reclassified from FNA-actionable to non-FNA-actionable, by TI-RADS category and nodule size.

Among Bethesda I/II nodules, 341 of 1119 guideline-actionable nodules were reclassified as non-FNA-actionable. Among Bethesda III/IV nodules, 97 of 397 were reclassified, and all 97 were BRAF*^V600E^*-negative. Surgical pathology was available for only 2 of these 97 nodules; 1 was malignant, and the remaining 95 lacked surgical verification. This observation documents malignancy within the reclassified indeterminate group, but the selected denominator of 2 cannot estimate the malignancy rate of all 97 nodules. Among the 2762 Bethesda V/VI nodules in the full cohort, 1920 were originally FNA-actionable, and 842 were not. Of the original 1920, 77 were reclassified to non-FNA status, and 1843 remained actionable. Adding 14 newly actionable Bethesda V/VI nodules gave 1857 in the final actionable group (Table 3; Figure 4). The 97 reclassified Bethesda III/IV nodules were distributed across TI-RADS 4B (51/97) and 4A (46/97), with 45/97 measuring 0.6–1.0 cm, 11/97 measuring 1.1–1.5 cm, and 41/97 measuring >1.5 cm; 88/97 were adjacent to the capsule or trachea.

Grade-stratified analysis showed that all reclassifications from FNA-actionable to non-FNA-actionable status occurred within TI-RADS 4A (276/301 originally FNA-actionable nodules) and TI-RADS 4B (242/956); no TI-RADS 4C or 5 nodules were reclassified, consistent with the protection of these categories from downgrade. In a sensitivity analysis restricted to nodules for which FNA was formally recommended, excluding those for which FNA could only be considered (*n* = 1813), the oracle BRAF*^V600E^*-informed strategy reduced FNA-actionable classifications by 267 nodules (14.7%) while retaining 790 of 810 Bethesda V/VI nodules (97.5%; 95% CI, 96.2–98.5%), consistent with the primary analysis, which additionally included nodules for which FNA could be considered.

The newly FNA-actionable group was small but included additional Bethesda V/VI nodules, reflecting escalation among BRAF*^V600E^*-positive nodules that did not initially meet guideline-based FNA criteria.

### 3.5. Surgical Pathology Subgroup

**Figure 5:**
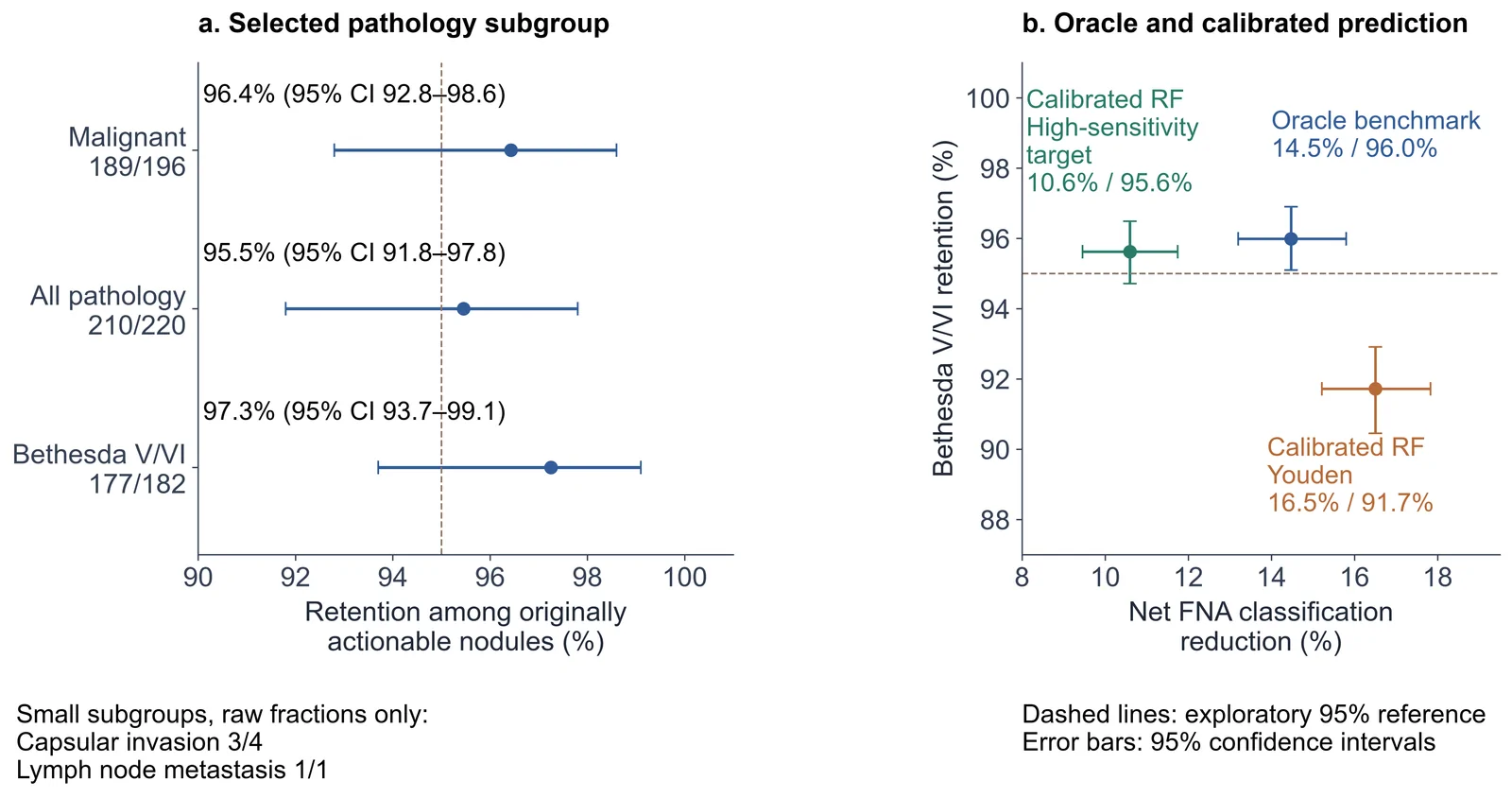
Selected surgical-pathology subgroup and patient-grouped calibrated prediction. (**a**) Retention among originally actionable nodules with surgical pathology, with exact 95% confidence intervals. Only 365/4944 nodules had pathology; the subgroup is subject to selection and partial-verification bias. Capsular-invasion and lymph-node-metastasis results are presented separately as raw fractions (3/4 and 1/1) and excluded from the percentage plot. (**b**) Net FNA classification reduction and Bethesda V/VI retention for the oracle rule and calibrated random forest predictions at inner-training-selected thresholds. Model estimates use untouched outer-test predictions; error bars show patient-cluster bootstrap 95% confidence intervals. The dashed 95% line is exploratory and has no validated safety interpretation. The high-sensitivity target refers to BRAF*^V600E^* sensitivity in inner training data.

Surgical pathology was available for 365/4944 nodules (7.4%). Among 196 histologically malignant nodules originally FNA-actionable, 189 remained actionable (96.4%; exact 95% CI, 92.8–98.6%), and 7 were reclassified to non-FNA status. This interval includes retention below the exploratory 95% reference. Among all 220 originally actionable nodules with pathology, 210 remained actionable (95.5%; 95% CI, 91.8–97.8%); this is retention of the pathology subgroup, not a composite of adverse endpoints. Bethesda V/VI retention within the pathology subgroup was 177/182 (97.3%; 95% CI, 93.7–99.1%). Retention was 3/4 for capsular invasion and 1/1 for lymph node metastasis. These very small subgroups are shown only as raw fractions and are excluded from the percentage plot (Table 3; Figure 5a).

### 3.6. Non-FNA Pathway and AS-Compatible Context

Under the BRAF*^V600E^*-informed strategy, 1990 nodules were assigned to the non-FNA-actionable pathway. These nodules were smaller than those remaining FNA-actionable, with a median maximum diameter of 6.0 mm versus 11.0 mm, and were more frequently remote from the thyroid capsule and trachea. Among non-FNA-actionable nodules, 719 met the predefined AS-compatible imaging and default clinical criteria (Figure 4).

### 3.7. Machine Learning Prediction and Decision Simulation

In patient-grouped five-fold cross-validation, random forest AUC was 0.693 (95% CI, 0.678–0.707), with average precision 0.585. AUCs were 0.672 for histogram gradient boosting, 0.697 for logistic regression, 0.702 for gradient boosting, and 0.695 for extra trees. Thus, random forest did not have the highest AUC in this comparison; it was retained as the reference model for decision simulation. The matched nodule-level random forest AUC was 0.698 (95% CI, 0.683–0.713), compared with 0.693 under patient grouping (paired difference, −0.006; 95% CI, −0.010 to −0.002). The grouped result is the primary estimate (Table 4).

Before calibration, grouped random forest predictions had a calibration intercept of −0.285, slope of 1.029, and Brier score of 0.219. Training-fold logistic recalibration yielded an outer-test calibration intercept of −0.008, slope of 0.965, and Brier score of 0.216, compared with 0.244 for the cohort-prevalence-only comparator. Calibrated AUC remained 0.693. These results indicate improved internal calibration, with discrimination remaining moderate; they do not establish transportability of probabilities or thresholds.

Using the calibrated random forest and inner-training-selected Youden thresholds, the simulation reduced actionable classifications by 570 (16.5%; 95% CI, 15.2–17.8%) to 2884, while retaining 1761/1920 Bethesda V/VI nodules (91.7%; 95% CI, 90.5–92.9%). With thresholds targeting at least 90% BRAF*^V600E^* sensitivity in inner training predictions, the reduction was from 366 (10.6%; 95% CI, 9.5–11.7%) to 3088, and Bethesda V/VI retention was 1836/1920 (95.6%; 95% CI, 94.7–96.5%); 84 originally actionable Bethesda V/VI nodules were reclassified to non-FNA status. Outer-test BRAF*^V600E^* sensitivity at this operating point was 89.6%. The Bethesda V/VI retention interval extended below 95%, so the point estimate does not establish a safety margin (Table 4; Figure 5b).

## 4. Discussion

### 4.1. Principal Findings

In this selected FNA-evaluated and molecularly tested cohort, guideline-based TI-RADS classified 69.9% of nodules as FNA-actionable, and 44.4% of these did not yield Bethesda V/VI cytology. Under the hypothetical assumption that BRAF*^V600E^* status was already known, the predefined reclassification rule reduced actionable classifications by a net 14.5% and retained 96.0% of originally actionable Bethesda V/VI nodules. These are retrospective classification outcomes. Although these findings do not establish that an initial biopsy can safely be avoided, they suggest that pre-biopsy molecular risk information may help refine risk stratification and identify nodules near ultrasound-based thresholds for more individualized management.

The rationale for studying molecular information is to assess whether it could refine management near ultrasound-based thresholds. However, an FNA that does not yield Bethesda V/VI may still provide clinically useful information, and Bethesda I/II cannot be treated as a uniform benign endpoint because Bethesda I is nondiagnostic. Likewise, indeterminate cytology may contain malignancy. We therefore interpret the reduction as a change in simulated FNA eligibility, while recognizing its potential clinical relevance for reducing low-yield FNA classifications and improving decision support, rather than directly equating it with the number of unnecessary procedures avoided.

### 4.2. Restrictions and Unresolved Safety of the Reclassification Rule

The predefined rule restricted downgrading to BRAF*^V600E^*-negative TI-RADS 4A/4B nodules and protected 4C/5 nodules. These constraints reflect the intended conservative design, but prospective validation is required to confirm clinical safety. BRAF*^V600E^* negativity cannot establish benignity, and 77 originally actionable Bethesda V/VI nodules were reclassified to non-FNA status. Bethesda V/VI retention is a cytological endpoint rather than a direct estimate of sensitivity for all malignancies. The exploratory 95% reference is not a validated noninferiority margin, and the malignant-subgroup confidence interval (92.8–98.6%) extends below it. Therefore, although the main cytological retention point estimate exceeded 95%, the safety of applying this rule in clinical decision-making requires further validation.

### 4.3. Sources of the Efficiency Gain

The reduction arose largely from reclassification of 341 Bethesda I/II and 97 Bethesda III/IV nodules, together with 77 Bethesda V/VI nodules and 3 with missing or unclear Bethesda classification. These distributions identify which categories contribute to the simulated reduction; they should not be interpreted as directly quantifying unnecessary FNA procedures avoided.

The Bethesda III/IV subgroup requires particular attention. Only 2 of the 97 reclassified nodules had surgical pathology, and 1 was malignant despite BRAF*^V600E^* negativity. The other 95 had no surgical verification. The observed fraction of 1/2 should neither be extrapolated as the malignancy rate of all 97 nor dismissed because of the small sample: it confirms that this downgrade rule can reclassify a malignant indeterminate nodule, while the risk among the unverified nodules remains unknown. Moreover, 88/97 were adjacent to the capsule or trachea, and 41/97 exceeded 1.5 cm. These findings highlight the need for systematic verification or longitudinal follow-up before applying the rule to indeterminate nodules in practice. The strict FNA-recommended sensitivity analysis (14.7% reduction; Bethesda V/VI retention 97.5%, 95% CI, 96.2–98.5%) addresses the eligibility definition, but cannot resolve this verification gap.

### 4.4. Surgical Pathology and Non-FNA Context

Surgical pathology was available for only 365/4944 nodules, and the decision to operate could depend on cytology, imaging, molecular findings, and clinical concerns. The surgical subgroup is therefore subject to selection and partial-verification bias and is not representative of the full cohort. Seven of 196 originally actionable malignant nodules were reclassified to non-FNA status. Retention of capsular-invasion and lymph-node-metastasis cases was 3/4 and 1/1, respectively; these fractions are insufficient to draw firm conclusions regarding uncommon adverse pathological features. Larger, systematically verified cohorts and follow-up of reclassified nodules are required to assess missed malignancy and its clinical consequences.

The AS-compatible analysis should be interpreted as contextual, not directive. FNA and active surveillance are not opposing management pathways; some nodules may undergo FNA and subsequently be managed with observation. In this study, AS-compatible status was used only to describe whether nodules assigned to the non-FNA-actionable pathway had features consistent with conservative management, including small size and absence of capsule or tracheal adjacency.

### 4.5. Clinical Implementability and Temporal Ordering of BRAF*^V600E^* Testing

The central limitation is temporal: BRAF*^V600E^* status generally comes from FNA-derived material and is unavailable before the initial biopsy decision. The oracle analysis is an optimistic benchmark for this specific rule under assumed molecular knowledge. It does not evaluate an available pre-biopsy test or demonstrate an observed reduction in performed procedures. Any future test must be evaluated with its own false-negative and false-positive results; its performance cannot be assumed to equal that of the oracle input.

The patient-grouped clinical-ultrasound model addresses within-patient overlap but remains an exploratory internal analysis. Random forest discrimination was moderate (AUC 0.693), and training-fold recalibration improved internal probability calibration without materially changing discrimination. At the training-selected high-sensitivity operating point, the simulated reduction was 10.6%, compared with 14.5% for the oracle benchmark, and Bethesda V/VI retention was 95.6% with a confidence interval extending below 95%. These findings do not establish a deployable cutoff or safety margin. External validation, assessment of calibration drift, and prospective evaluation of the full diagnostic pathway remain necessary.

Blood-based liquid biopsy is one possible route to obtaining a molecular signal before thyroid FNA. However, blood collection is minimally invasive, and current evidence does not establish a reliable rule-out test. Assays based on digital PCR or sequencing may detect circulating tumor DNA carrying BRAF*^V600E^*, but detection varies with assay method and disease burden. Jensen et al. detected circulating BRAF*^V600E^* in 24/57 patients with tissue-mutant papillary thyroid cancer, including only 4/19 in the low-risk group [22]. Condello et al. detected no corresponding circulating mutation among 22 tissue-mutant cancers [23]. In a preoperative series without distant metastasis, Sato et al. detected the mutation in plasma in 5/16 patients whose primary tumors carried BRAF*^V600E^* [24]. These studies support biological feasibility while illustrating limited sensitivity, particularly relevant to small or localized nodules.

A negative plasma assay may reflect low tumor-DNA shedding or an assay detection limit, rather than absence of a tumor mutation; a tumor may also be malignant through a non-BRAF*^V600E^* pathway. Moreover, a blood signal cannot by itself identify the responsible nodule in a patient with multiple nodules. A clinically testable extension would combine a validated pre-FNA assay with TI-RADS in a prospectively enrolled, unselected biopsy-decision cohort, with prespecified thresholds, nodule-level matching, and pathological or adequate longitudinal verification of reclassified nodules. Assay reproducibility, mutation-negative cancers, and missed clinically significant malignancy would need explicit evaluation. Thus, circulating BRAF*^V600E^* testing remains a promising but investigational approach for informing pre-biopsy risk stratification.

### 4.6. Limitations

Selection into this cohort can affect the magnitude and direction of the simulated 14.5% reduction. A high proportion of TI-RADS 4C nodules, which are protected from downgrading, and a high BRAF*^V600E^*-positive prevalence could limit opportunities for de-escalation. Conversely, selecting already biopsied intermediate-risk nodules near size or location thresholds could increase the fraction eligible for the downgrade rule. The net effect also depends on newly actionable upgrades and on the baseline actionable denominator. It cannot be inferred from mutation prevalence alone. Because no unselected pre-biopsy comparison cohort was available, we cannot determine whether 14.5% overestimates or underestimates the effect in that setting, and the same reduction should not be expected without external testing.

Other limitations include the retrospective design, incomplete guideline modifiers, and reliance on a Bethesda endpoint for most nodules. Patient grouping reduces within-patient overlap during internal validation but does not address referral selection, temporal change, or transportability to other centers. Although the oracle rule was defined before outcome inspection, its effect was evaluated in one selected cohort without prospective testing. The machine-learning pipelines used fixed analysis settings, and uncertainty intervals were conditioned on the generated out-of-fold predictions. Neither an external validation dataset nor prospective outcomes were available. Sparse and selective surgical verification, particularly in the reclassified Bethesda III/IV group, limits conclusions regarding the clinical safety of biopsy deferral.

## 5. Conclusions

Under the hypothetical assumption that BRAF*^V600E^* status is known before FNA, the predefined rule reduced simulated FNA-actionable classifications by 14.5% in a selected, molecularly tested cohort while retaining 96.0% of originally actionable Bethesda V/VI nodules. Patient-grouped, calibrated prediction reproduced a smaller simulated reduction and requires external validation. These findings support further investigation of pre-biopsy risk information, but do not support using BRAF*^V600E^* negativity to defer an indicated biopsy or implementing the present rule in clinical practice.

## Figures and Tables

**Table 1:** Baseline clinical, ultrasound, TI-RADS, cytological, molecular, and FNA recommendation characteristics of thyroid nodules stratified by BRAF*^V600E^* status.

Characteristic	Overall	BRAF*^V600E^*-Negative	BRAF*^V600E^*-Positive
Clinical characteristics			
Age, years, median (IQR)	41.0 (34.0–53.0)	43.0 (35.0–55.0)	39.0 (33.0–49.0)
Female sex, *n* (%)	3865 (78.2%)	2305 (80.9%)	1560 (74.5%)
Ultrasound and location features			
Maximum nodule diameter, mm, median (IQR)	9.0 (6.0–14.0)	9.5 (6.0–18.0)	8.0 (6.0–11.0)
Solid or predominantly solid, *n* (%)	4260 (86.2%)	2285 (80.2%)	1975 (94.3%)
Hypoechoic or markedly hypoechoic, *n* (%)	4486 (90.7%)	2462 (86.4%)	2024 (96.6%)
Irregular margin, *n* (%)	4337 (87.7%)	2410 (84.6%)	1927 (92.0%)
Punctate echogenic foci or microcalcifications, *n* (%)	1725 (34.9%)	815 (28.6%)	910 (43.4%)
Vertical orientation, *n* (%)	3306 (66.9%)	1735 (60.9%)	1571 (75.0%)
Capsule adjacent/protrusion, *n* (%)	3707 (75.0%)	2105 (73.9%)	1602 (76.5%)
Trachea adjacent, *n* (%)	1298 (26.3%)	760 (26.7%)	538 (25.7%)
TI-RADS category			
TI-RADS 2, *n* (%)	0 (0.0%)	0 (0.0%)	0 (0.0%)
TI-RADS 3, *n* (%)	56 (1.1%)	53 (1.9%)	3 (0.1%)
TI-RADS 4A, *n* (%)	402 (8.1%)	352 (12.4%)	50 (2.4%)
TI-RADS 4B, *n* (%)	1180 (23.9%)	828 (29.1%)	352 (16.8%)
TI-RADS 4C, *n* (%)	3300 (66.7%)	1614 (56.6%)	1686 (80.5%)
TI-RADS 5, *n* (%)	6 (0.1%)	2 (0.1%)	4 (0.2%)
Bethesda cytology			
Bethesda 1, *n* (%)	275 (5.6%)	267 (9.4%)	8 (0.4%)
Bethesda 2, *n* (%)	1272 (25.7%)	1247 (43.8%)	25 (1.2%)
Bethesda 3, *n* (%)	418 (8.5%)	360 (12.6%)	58 (2.8%)
Bethesda 4, *n* (%)	193 (3.9%)	185 (6.5%)	8 (0.4%)
Bethesda 5, *n* (%)	743 (15.0%)	370 (13.0%)	373 (17.8%)
Bethesda 6, *n* (%)	2019 (40.8%)	399 (14.0%)	1620 (77.3%)
TI-RADS guideline pathway			
Guideline FNA-actionable, *n* (%)	3454 (69.9%)	1964 (69.0%)	1490 (71.1%)
AS-compatible by corrected guideline data, *n* (%)	737 (14.9%)	439 (15.4%)	298 (14.2%)

Note: TI-RADS, Chinese Thyroid Imaging Reporting and Data System; FNA, fine-needle aspiration; IQR, interquartile range. Continuous variables are median (IQR); categorical variables are *n* (%).

**Table 2:** TI-RADS biopsy recommendation efficiency gap and BRAF*^V600E^*-informed decision impact.

Strategy (Oracle BRAF*^V600E^* Status)	New FNA *n*	Net FNA Reduction *n*	Net FNA Reduction %	Newly FNA-Actionable *n*	Bethesda V/VI Yield Among FNA %	Original-FNA Bethesda V/VI Capture %	Missed Original-FNA Bethesda V/VI *n*	Added Observation Bethesda V/VI *n*
Guideline TI-RADS	3454	0	0	0	55.6	100	0	0
Main BRAF*^V600E^*-informed TI-RADS Strategy	2954	500	14.5	18	62.9	96	77	14

Note: Under the TI-RADS guidelines, 3454 of 4944 nodules (69.9%) were FNA-actionable, including 1813 recommended for FNA and 1641 nodules for which FNA may be considered; 44.4% of FNA-actionable nodules did not yield Bethesda V/VI cytology. The main strategy (using oracle, i.e., already-known BRAF*^V600E^* status) was mutation-positive upgrade plus mutation-negative TI-RADS 4A/4B downgrade. For the main strategy, 518 originally FNA-actionable nodules were reclassified to the non-FNA-actionable pathway, whereas 18 nodules became newly FNA-actionable, resulting in a net reduction of 500 FNA-actionable recommendations. TI-RADS 4C/5 nodules were not downgraded. For the main strategy, patient-cluster bootstrap 95% CIs were: absolute reduction, 10.1 percentage points (95% CI, 9.2–11.0); relative reduction, 14.5% (95% CI, 13.2–15.8%); Bethesda V/VI capture, 96.0% (95% CI, 95.1–96.9%).

**Table 3:** Cytology-specific reclassification and descriptive surgical-pathology retention under the main oracle BRAF*^V600E^*-informed strategy.

Analysis Group	Full-Cohort Total or Endpoint-Positive *n*	Initially FNA-Actionable *n*	Final Actionable *n* or Pathology Cases Retained	Reclassified to Non-FNA *n*	Key Result
Bethesda I/II	1547	1119	781	341	Reduction 30.5%; BRAF*^V600E^*-negative among reclassified 341
Bethesda III/IV	611	397	301	97	Reduction 24.4%; BRAF*^V600E^*-negative among reclassified 97
Bethesda V/VI	2762	1920	1857	77	Reduction 4.0%; BRAF*^V600E^*-negative among reclassified 77
Bethesda missing/unclear	24	18	15	3	Reduction 16.7%; BRAF*^V600E^*-negative among reclassified 3
Surgical pathology subgroup	365	220	210	10	Capture 95.5%
Histologically malignant disease	333	196	189	7	Capture 96.4%
Capsular invasion	6	4	3	1	Retained 3/4
Lymph node metastasis	2	1	1	0	Retained 1/1
Bethesda V/VI in pathology subgroup	306	182	177	5	Capture 97.3%

Note: Total Bethesda counts refer to all 4944 nodules; guideline FNA counts refer only to the 3454 initially actionable nodules. For Bethesda V/VI, 2762 = 1920 initially actionable + 842 initially non-actionable; the final actionable count is 1857 = 1843 retained + 14 newly actionable. Retention uses 1843/1920, whereas yield uses 1857/2954. For cytology rows, final actionable counts include newly actionable nodules, so they need not equal the baseline count minus gross reclassifications. Pathology rows show retention among initially actionable nodules within the selected surgical subgroup, not cohort-wide diagnostic sensitivity. Exact 95% CIs are 92.8–98.6% for malignant retention, 91.8–97.8% for all pathology-subgroup retention, and 93.7–99.1% for Bethesda V/VI retention. Capsular invasion and lymph node metastasis were reported in 3/4 and 1/1 cases, respectively. Surgical pathology was available for 2/97 reclassified Bethesda III/IV nodules, and 1/2 was malignant.

**Table 4:** Patient-grouped model evaluation and exploratory decision simulation after training-fold recalibration.

Model or Strategy	AUC	BRAF Sensitivity %	BRAF Specificity %	FNA Reduction %	V/VI Retention %	Details
Random forest	0.693	69.7	58.4	—	—	Average precision 0.585
Histogram gradient boosting	0.672	53.8	68.4	—	—	Average precision 0.562
Logistic regression	0.697	52.4	72.6	—	—	Average precision 0.580
Gradient boosting	0.702	55.8	72.0	—	—	Average precision 0.598
Extra trees	0.695	74.0	55.1	—	—	Average precision 0.586
Oracle benchmark	—	—	—	14.5	96.0	2954 actionable; 500 net reduction; 77 V/VI reclassified
Calibrated random forest Youden threshold	0.693	65.8	62.3	16.5	91.7	2884 actionable; 570 net reduction; 159 V/VI reclassified
Calibrated random forest High-sensitivity target	0.693	89.6	34.0	10.6	95.6	3088 actionable; 366 net reduction; 84 V/VI reclassified

Note: The first five rows report raw-probability model performance from the same five patient-grouped outer folds, with sensitivity/specificity at 0.50. Random forest was retained for decision simulation; it was not the highest-AUC model in this comparison. All preprocessing was fitted within training folds. Decision rows use random forest probabilities after logistic recalibration, with calibration and thresholds learned from four-fold patient-grouped inner predictions only. The high-sensitivity target was at least 90% for BRAF*^V600E^* in inner training data; achieved outer-test sensitivity was 89.6%. Calibrated random forest AUC was 0.693 (95% CI, 0.678–0.707), Brier score 0.216, calibration intercept −0.008, and slope 0.965. The prevalence-only Brier comparator was 0.244. These results provide exploratory internal validation, not an externally validated fixed cutoff or evidence of safe biopsy deferral. The high-sensitivity Bethesda V/VI retention CI was 94.7–96.5%, crossing below the unvalidated 95% reference. Oracle and model rows both describe hypothetical classification changes, not observed procedures avoided.

## Data Availability

The data presented in this study are not publicly available because they contain protected clinical information and are subject to institutional and ethical restrictions. De-identified data may be made available from the corresponding author upon reasonable request and subject to approval by China-Japan Friendship Hospital and the relevant ethics committee.

## Abbreviations

AS, active surveillance; TI-RADS, Thyroid Imaging Reporting and Data System; FNA, fine-needle aspiration; IQR, interquartile range; ML, machine learning.
